# Type 2 diabetes and postoperative pneumonia: An observational, population-based study using the Spanish Hospital Discharge Database, 2001-2015

**DOI:** 10.1371/journal.pone.0211230

**Published:** 2019-02-06

**Authors:** Ana López-de-Andrés, Napoleon Perez-Farinos, Javier de Miguel-Díez, Valentín Hernández-Barrera, Isabel Jiménez-Trujillo, Manuel Méndez-Bailón, José M. de Miguel-Yanes, Rodrigo Jiménez-García

**Affiliations:** 1 Preventive Medicine and Public Health Teaching and Research Unit. Health Sciences Faculty, Rey Juan Carlos University, Alcorcón, Madrid, Spain; 2 Public Health and Psychiatry Department, Faculty of Medicine, Universidad de Malaga, Malaga, Spain; 3 Respiratory Department, Hospital General Universitario Gregorio Marañón, Facultad de Medicina, Universidad Complutense de Madrid (UCM), Instituto de Investigación Sanitaria Gregorio Marañón (IiSGM), Madrid, Spain; 4 Internal Medicine Department, Hospital Universitario Clínico San Carlos, Madrid, Spain; 5 Internal Medicine Department, Hospital General Universitario Gregorio Marañón, Madrid, Spain; University of Notre Dame Australia, AUSTRALIA

## Abstract

**Purpose:**

We analyzed temporal trends, demographic and clinical characteristics and hospital mortality rates of postoperative pneumonia among type 2 diabetes mellitus (T2DM) patients in Spain from 2001 to 2015. We also compared the incidence, comorbidities and mortality between patients with and without T2DM suffering from postoperative pneumonia. Finally, we analyzed the factors involved in the prediction of in-hospital mortality among patients suffering postoperative pneumonia.

**Methods:**

We used the Spanish National Hospital Discharge Database for the period 2001–2015. We analyzed patients aged 40 years or over who had been hospitalized for a surgical procedure and suffered pneumonia or ventilator-associated pneumonia during their hospital admission. We compared patients with and without T2DM. The main outcome measures were the type of surgical procedure, the presence of a comorbidity, the type of isolated pathogens, admission to the emergency room (ER) and in-hospital mortality (IHM).

**Results:**

We selected 117,665 hospitalized patients who suffered postoperative pneumonia (16.9% with T2DM). After multivariable adjustment, T2DM patients had a 21% higher incidence of postoperative pneumonia than nondiabetic patients (IRR 1.21, 95% CI 1.03–1.42). The IHM was approximately 31% in both groups. Predictors of IHM included age, the presence of comorbidities, treatment with a pleural drainage tube, dialysis, blood transfusion, mechanical ventilation and admission to the ER. From 2001 to 2015, the IHM decreased significantly in both populations. Suffering from T2DM was not a predictor of IHM (OR 0.99, 95% CI 0.96–1.03) in our investigation.

**Conclusions:**

T2DM patients have a higher incidence of postoperative pneumonia than those without this disease. The IHM decreased from 2001 to 2015, regardless of T2DM status. T2DM did not predict a higher IHM after suffering from postoperative pneumonia.

## Introduction

Hospital-acquired pneumonia (HAP) represents approximately 15% of all infections acquired during hospitalizations [1]. Over the last two decades, research has focused mainly on ventilator-associated pneumonia (VAP), and as a consequence, the incidence of this complication has decreased and patient outcomes have improved [2–5]. In Spain, a recent investigation showed that the incidence rates of VAP improved significantly from 41.7 cases/100,000 inhabitants in 2010 to 40.55 cases/100,000 inhabitants in 2014 [6]. However, VAP is not the only form of HAP; a recent report with data from the US National Inpatient Sample dataset found that nonventilator HAP was associated with mortality, a greater length of hospital stay and an increase in the cost of care [7].

Kazaure HS et al. analyzed complications for all surgical procedures, finding that postoperative pneumonia is very frequent, representing the third most common complication and resulting in increased morbidity and mortality of postsurgical patients [8].

Diabetes is a risk factor that contributes to the development of postoperative pneumonia for several surgical specialties [9]. In general, surgery, cardiothoracic surgery, orthopedic surgery and spine surgery, diabetes was associated with an approximately 2-fold higher risk of postoperative pneumonia [10–13]. Poor glycemic control and a longer duration of diabetes were associated with increased susceptibility to hospital-acquired infections [14].

In this study, we analyzed temporal trends, demographic and clinical characteristics and hospital mortality of postoperative pneumonia among type 2 diabetes mellitus (T2DM) patients in Spain from 2001 to 2015. We also compared the incidence, comorbidities and mortality between patients with and without T2DM suffering postoperative pneumonia. Finally, we tested which factors predicted in-hospital mortality among patients suffering postoperative pneumonia.

## Materials and methods

The Spanish National Hospital Discharge Database was used to conduct this observational retrospective investigation. The Spanish National Hospital Discharge Database (CMBD, *Conjunto Mínimo Básico Datos*) was implemented in Spain in 1987. According to the Spanish legislation, all hospitals must submit information of every single patient hospitalized for at least one night in a hospital ward to the Ministry of Health. The variables included were dates of admission and discharge, age, sex, hospitalization services, type of admission (emergency room/scheduled), hospital size and discharge status (discharged home, discharged and transferred to other facilities, dead, not stated or not reported). Medical diagnoses (up to 14) and procedures (up to 20) conducted during hospitalization were obtained from the discharge report. The first or primary diagnosis was the disease that was considered responsible for the hospital admission of the patient after investigation [15]. The International Classification of Diseases-Ninth Revision, Clinical Modification (ICD-9-MC) was used for coding in the CMBD.

We analyzed all registries included in the CMBD from 2001 to 2015. For study purpose, only subjects aged ≥40 years who had an ICD-9-MC code for pneumonia (507.xx, 480.xx-488.xx) or VAP (997.31) in any of their secondary diagnosis fields were included. Following the recommendations of Guevara et al., if a patient’s primary diagnosis was bacteremia (790.7), sepsis (0.38, 995.92, 995.91), meningitis (322.xx). empyema (510.9, 510.0) or pneumonia (997.31, 507.xx, 480.xx-488.xx), they were excluded as these patients may suffer from community-acquired pneumonia [16].

Patients who presented codes 250.x0 or 250.x2 in any of the diagnosis positions were considered T2DM sufferers. Those with codes 250.x1 or 250.x3 were considered type 1 diabetes mellitus sufferers and excluded. The remaining patients were considered nondiabetic subjects.

The presence of comorbidities was measured using the Charlson comorbidity index (CCI) [17].

We categorized the surgical procedures conducted during hospitalization using the following ICD-9-CM codes in any of the 20 procedure fields: i) codes 01–05 (surgeries involving the nervous system); ii) codes 30–34 (surgeries involving the respiratory system); iii) codes 35–39 (surgeries involving the cardiovascular system); iv) codes 42–54 (surgeries involving the digestive system); v) codes 55–75 (surgeries involving the urinary system/male and female genital organs/obstetrical procedures); and iv) codes 76–89 (surgeries involving the musculoskeletal system/integumentary system).

Other procedures also identified included dialysis (codes 39.95, 54.98), transfusion (codes 99.00, 99.01–99.08), thoracentesis (code 34.91), invasive mechanical ventilation (codes 96.7, 96.70–72), pleural drainage tube (codes 34.0, 34.01–34.09), noninvasive mechanical ventilation (code 93.90) and respiratory culture (codes 90.4x).

The pneumonia pathogens analyzed in our study have been described by de Miguel-Díez J et al. [6].

Finally, for each patient, hospital admission (emergency room or programmed admission) and patient death during hospitalization (IHM) were analyzed.

### Statistical analysis

To assess time trends, three periods were considered: 2001 to 2005, 2006 to 2010 and 2011 to 2015.

To assess the temporal trends, we estimated incidence rates by dividing those patients who suffered postoperative pneumonia by the total number of those undergoing a surgical procedure. These incidences were calculated according to the presence or absence of T2DM and are expressed per 100,000 patients based on surgical procedure. Poisson regression models adjusted by “year of admission” (three periods included as continuous variables) and “rate of hospital admissions adjusted by age and sex” were constructed to assess time trends.

In the model including the total population, “T2DM” was also included as a covariate. In this last model, we included the interaction “year*T2DM”. The interaction was not significant. As we included the rate of hospital admissions adjusted by sex and age, we did not include more interactions.

Descriptive statistics included proportions (categorical variables) and means with standard deviations (continuous variables). Comparisons of proportions were performed using the χ^2^ test and ANOVA or Student’s *t*-test for means.

Logistic regression models were constructed to assess changes over time in the categorical variables and to identify predictors of IHM.

The construct of the multivariable logistic regression models for IHM followed the steps recommended by El-Amrani-Joutey et al. [18].

Statistical analyses were performed with Stata 10.1 (Stata, College Station, Texas, USA). Statistical significance was set at p<0.05 (2-tailed).

### Ethical aspects

In this investigation, data were provided to the authors anonymized so it was impossible to identify patients. According to Spanish legislation, given the characteristics of the database and as the Spanish Ministry of Health considered the study ethically acceptable, it was not necessary to obtain the approval of an ethics committee.

### Patient and public involvement

For this investigation, we used a publicly accessible, anonymous and mandatory dataset, so patients’ priorities, experience, and preferences were not considered in the development of the research question and outcome measures. Furthermore, patients were not involved in the design or recruitment of this study. Finally, as we do not have personal data, we cannot disseminate the results of the study to participants.

## Results

We analyzed data from 117,665 hospitalizations of patients ≥40 years who suffered postoperative pneumonia in Spain from 2001 to 2015. T2DM was a diagnosis codified in 16.9% of patients (n = 20,003).

The incidence, comorbidities and type of surgical procedures conducted among patients who suffered postoperative pneumonia according to the presence of T2DM are shown in Table 1.

**Table 1:** Sociodemographic and clinical characteristics of hospitalized patients who suffered from postsurgical pneumonia in Spain from 2001 to 2015 according to diabetes status.

		2001–05		2006–10		2011–15		Total	
		Type 2 diabetes	No diabetes	Type 2 diabetes	No diabetes	Type 2 diabetes	No diabetes	Type 2 diabetes	No diabetes
Number of postsurgical pneumonia cases		4978	28468	7074	33576	7951	35618	20003	97662
Incidence per 100,000 surgeries*ǂ ^a^^,^ ^b^^,^ ^c^^,^ ^d^		393.67	314.46	418.77	318.02	419.28	300.52	397.35	310.40
Female sex, n (%)*ǂ		1773(35.62)	9012(31.66)	2464(34.83)	10810(32.2)	2632(33.1)	11879(33.35)	6869(34.34)	31701(32.46)
Age, mean (SD) *ǂ ^a^^,^ ^b^^,^ ^c^^,^ ^d^		72.07(10,56)	68.3(13.18)	72.73(10.78)	68.78(13.56)	73.86(11.03)	70.19(13.63)	73.01(10.85)	69.15(13.5)
CCI, mean (SD) *ǂ ^a^^,^ ^b^^,^ ^c^^,^ ^d^		1.27(0.96)	1.07(0.9)	1.29(0.97)	1.11(0.91)	1.37(1.01)	1.19(0.94)	1.32(0.98)	1.13(0.92)
CCI, n (%) ^a^^,^ ^b^^,^ ^c^^,^ ^d^	0* ǂ	1100(22.1)	8118(28.52)	1484(20.98)	9026(26.88)	1543(19.41)	8782(24.66)	4127(20.63)	25926(26.55)
	1* ǂ	2050(41.18)	12454(43.75)	2960(41.84)	14626(43.56)	3195(40.18)	15134(42.49)	8205(41.02)	42214(43.22)
	≥2* ǂ	1828(36.72)	7896(27.74)	2630(37.18)	9924(29.56)	3213(40.41)	11702(32.85)	7671(38.35)	29522(30.23)
Surgeries involving the nervous system * ^a^^,^ ^b^^,^ ^c^^,^ ^d^		532(10.69)	3979(13.98)	777(11.01)	4415(13.17)	517(6.54)	2681(7.57)	1826(9.16)	11075(11.37)
Surgeries involving the respiratory system * ǂ ^a^^,^ ^b^^,^ ^c^^,^ ^d^		1147(23.04)	7829(27.5)	1281(18.16)	7878(23.51)	791(10.01)	4466(12.61)	3219(16.15)	20173(20.71)
Surgeries involving the cardiovascular system * ǂ ^a^^,^ ^b^^,^ ^c^^,^ ^d^		1230(24.71)	4986(17.51)	1322(18.74)	4960(14.8)	1248(15.8)	4848(13.69)	3800(19.06)	14794(15.19)
Surgeries involving the digestive system * ǂ ^a^^,^ ^b^^,^ ^c^^,^ ^d^		824(16.55)	5808(20.4)	1160(16.44)	6749(20.14)	1606(20.33)	9749(27.53)	3590(18.01)	22306(22.9)
Surgeries involving the urinary system/male and female genital organs/obstetrical procedures * ǂ ^a^^,^ ^b^^,^ ^c^^,^ ^d^		162(3.25)	893(3.14)	254(3.6)	1118(3.34)	324(4.1)	1539(4.35)	740(3.71)	3550(3.64)
Surgeries involving the musculoskeletal system/integumentary system* ǂ ^a^^,^ ^b^^,^ ^c^^,^ ^d^		845(16.97)	3267(11.48)	1323(18.75)	3942(11.76)	1893(23.96)	6290(17.76)	4061(20.37)	13499(13.86)

Incidence per 100,000 surgeries: Incidence calculated over the total number of hospitalizations by surgical specialty in Spain that year.

CCI: Charlson comorbidity index.

* p value for time trends among nondiabetic patients using Poisson or logistic regression adjusted by age and sex when appropriate.

^ǂ^ p value for time trends among diabetic patients using Poisson or logistic regression adjusted by age and sex when appropriate.

^a,b,c, d^p value for differences when comparing patients with and without diabetes using the χ2 test (proportions) and Student’s *t*-test (means), as appropriate for; ^a^ period 2001–2005; ^b^ period 2006–2010; ^c^ period 2011–2015 and ^d^ period 2001–2015.

The overall incidence of postoperative pneumonia per 100,000 surgeries was significantly higher in T2DM patients than in non-T2DM patients (397.35 vs. 310.40 cases; p<0.001); these differences were also significant in all time periods analyzed. The incidence of postoperative pneumonia rose significantly from 393.67 cases per 100,000 surgeries in patients with T2DM in 2001–05 to 419.28 cases per 100,000 surgeries in 2011–15 (p<0.001). However, in patients without diabetes, the incidence of postoperative pneumonia decreased significantly, as shown in Table 1 (p<0.001).

According to the adjusted Poisson regression analysis, patients with T2DM had a 21% higher incidence of postoperative pneumonia than those without diabetes (IRR, 1.21 95% CI 1.03–1.42).

Postoperative pneumonia was identified more frequently among men than women in both populations studied (65.66% and 67.54% for T2DM and nondiabetic patients, respectively p<0.001). The percentage of males affected increased significantly (p<0.001) in patients with T2DM; however, it decreased in those without diabetes (p<0.001). The mean age was significantly higher in those with T2DM (73.01±10.85 years vs. 69.15±13.5 years; p<0.001), and they also had a higher mean CCI index (1.32±0.98 vs 1.13±0.92; p<0.001). The mean age and CCI increased significantly over time among T2DM patients.

In patients with T2DM, the most frequent surgical procedures were surgeries of the musculoskeletal system/integumentary system (20.37%), followed by surgeries involving the cardiovascular system (19.06%) and the digestive system (18.01%). T2DM patients included in our investigation had undergone significantly more frequent obstetrical surgeries of the female reproductive organs and surgeries of the male urinary system, cardiovascular system, musculoskeletal system and integumentary system than nondiabetic patients (Table 1).

Diagnostic and therapeutic procedures, isolated pathogens and in-hospital outcomes for patients who suffered postsurgical pneumonia according to diabetes status are shown in Table 2.

**Table 2:** Procedures, hospital outcomes and isolated pathogens of hospitalized patients who suffered from postsurgical pneumonia in Spain from 2010 to 2015 according to diabetes status.

	2001–05		2006–10		2011–15		Total	
	Type 2 diabetes	No diabetes	Type 2 diabetes	No diabetes	Type 2 diabetes	No diabetes	Type 2 diabetes	No diabetes
Nonmechanical ventilation, n (%)* ǂ ^a, b, c, d^	146(2.93)	672(2.36)	290(4.1)	1637(4.88)	630(7.92)	3161(8.87)	1066(5.33)	5470(5.6)
Mechanical ventilation, n (%)* ǂ ^a, b, c, d^	1592(31.98)	10512(36.93)	2129(30.1)	13037(38.83)	2043(25.69)	12535(35.19)	5764(28.82)	36084(36.95)
Thoracocentesis, n (%)* ^a, b, c, d^	102(2.05)	735(2.58)	152(2.15)	895(2.67)	150(1.89)	1038(2.91)	404(2.02)	2668(2.73)
Pleural drainage tube, n (%)* ^a, b, c, d^	187(3.76)	1625(5.71)	272(3.85)	2215(6.6)	267(3.36)	2198(6.17)	726(3.63)	6038(6.18)
Transfusion, n (%) * ǂ ^a, b, c, d^	977(19.63)	6526(22.92)	1481(20.94)	7905(23.54)	1747(21.97)	8773(24.63)	4205(21.02)	23204(23.76)
Dialysis, n (%)* ^c^^,^ ^d^	251(5.04)	1353(4.75)	365(5.16)	1916(5.71)	398(5.01)	2174(6.1)	1014(5.07)	5443(5.57)
*Pseudomonas*, n (%) * ǂ ^a, b, c, d^	203(4.08)	1660(5.83)	245(3.46)	2013(6)	221(2.78)	1582(4.44)	669(3.34)	5255(5.38)
*Staphylococcus aureus*, n (%) * ǂ ^a, b, c, d^	231(4.64)	1524(5.35)	255(3.6)	1537(4.58)	172(2.16)	1112(3.12)	658(3.29)	4173(4.27)
*Streptococcus pneumoniae*, n (%)* ǂ	388(7.79)	2182(7.66)	522(7.38)	2346(6.99)	262(3.3)	1235(3.47)	1172(5.86)	5763(5.9)
*Haemophilus influenzae*, n (%)* ǂ ^b^^,^ ^c^^,^ ^d^	72(1.45)	491(1.72)	78(1.1)	516(1.54)	69(0.87)	475(1.33)	219(1.09)	1482(1.52)
Aspergillosis, n (%)* ^a, b, c, d^	28(0.56)	247(0.87)	33(0.47)	292(0.87)	55(0.69)	425(1.19)	116(0.58)	964(0.99)
Miscellaneous, n (%)* ǂ ^b^^,^ ^c^^,^ ^d^	92(1.85)	555(1.95)	103(1.46)	717(2.14)	99(1.25)	761(2.14)	294(1.47)	2033(2.08)
Respiratory culture, n (%)* ^b^^,^ ^c^^,^ ^d^	163(3.27)	969(3.4)	228(3.22)	1320(3.93)	239(3.01)	1469(4.12)	630(3.15)	3758(3.85)
Emergency room admission, n (%)* ^a, b, c, d^	3868(77.7)	20767(72.95)	5418(76.59)	24616(73.31)	6063(76.25)	26251(73.7)	15349(76.73)	71634(73.35)
In-hospital mortality, n (%)* ǂ	1791(35.98)	9904(34.79)	2307(32.61)	10691(31.84)	2114(26.59)	9828(27.59)	6212(31.06)	30423(31.15)

* p value for time trends among nondiabetic patients using Poisson or logistic regression adjusted by age and sex when appropriate.

^ǂ^ p value for time trends among diabetic patients using Poisson or logistic regression adjusted by age and sex when appropriate.

^a,b,c, d^p value for differences when comparing patients with and without diabetes using the χ2 test (proportions) and Student’s *t*-test (means), as appropriate for; ^a^ period 2001–2005; ^b^ period 2006–2010; ^c^ period 2011–2015 and ^d^ period 2001–2015.

Mechanical ventilation (28.82%) and transfusion (21.02%) were the procedures most frequently performed among T2DM patients. However, thoracentesis, dialysis (p<0.001), mechanical ventilation (p<0.001), transfusion (p<0.001), and pleural drainage tubes (p<0.001) were more frequently codified among nondiabetic patients.

Invasive mechanical ventilation was used in a smaller proportion of patients over time, whereas transfusion and noninvasive mechanical ventilation were more frequently used over time (both p<0.001).

Among patients with T2DM, the most commonly identified pathogens were *Streptococcus pneumoniae* (5.86%), *Pseudomonas* (3.34%) and *Staphylococcus aureus* (3.29%). The prevalence of the pathogens analyzed (except *Streptococcus pneumoniae*) was higher in patients without diabetes than in those with T2DM (p<0.001). For both groups of patients, the prevalence of all pathogens decreased from 2001 to 2015 (p<0.001).

Respiratory cultures were more frequently performed in those without T2DM (3.85%) than in those with the disease (3.15%) (p = 0.002).

The proportion of patients admitted through the emergency room was higher in patients with T2DM (76.73% vs 73.35%, p<0.001).

Over the fifteen years, IHM was approximately 31% in both groups. However, crude IHM decreased over time from 35.98% to 26.59% in patients with T2DM (p<0.001), and from 34.79% to 27.59% in those without diabetes (p<0.001).

Table 3 and Table 4 show the characteristics of the hospitalizations during which patients with and without T2DM developed postsurgical pneumonia according to hospital survival.

**Table 3:** Sociodemographic and clinical characteristics of hospitalized patients with and without diabetes who suffered from postsurgical pneumonia according to hospitalization survival in Spain, 2001–2015.

		In-hospital mortality		P value
		Type 2 diabetes	No diabetes	
Sex	Male, n (%)	4020(30.61)	20646(31.3)	0.118
	Female, n (%)	2192(31.91)	9777(30.84)	0.082
Age groups (years) ^a^^,^ ^b^	40–54, n (%)	277(21.57)	3531(20.77)	0.495
	55–64, n (%)	780(26.62)	4491(26.17)	0.610
	65–74, n (%)	1779(30.04)	8105(33.17)	<0.01
	75–84, n (%)	2403(33.67)	9790(36.32)	<0.01
	≥ 85, n (%)	973(35.64)	4506(37.2)	0.127
CCI, n (%) ^a^^,^ ^b^	0	1076(26.07)	6706(25.87)	0.779
	1	2553(31.12)	13278(31.45)	0.545
	≥2	2583(33.67)	10439(35.36)	0.006
Surgeries involving the nervous system, n (%)^a^^,^ ^b^		759(41.57)	3797(34.28)	<0.01
Surgeries involving the respiratory system, n (%)^a^^,^ ^b^		1262(39.2)	7420(36.78)	0.008
Surgeries involving the cardiovascular system, n (%)^a^^,^ ^b^		918(24.16)	4304(29.09)	<0.01
Surgeries involving the digestive system, n (%)^a^^,^ ^b^		1101(30.67)	6818(30.57)	0.901
Surgeries involving the urinary system/male and female genital organs/obstetrical procedures, n (%)^a^^,^ ^b^		182(24.59)	773(21.77)	0.094
Surgeries involving the musculoskeletal system/integumentary system, n (%)^a^^,^ ^b^		1244(30.63)	3756(27.82)	0.001

CCI: Charlson comorbidity index.

p value for the difference when comparing patients with and without diabetes.

^a^ Significant association of the study variable with IHM among nondiabetic patients.

^b^ Significant association of the study variable with IHM among type 2 diabetes patients.

**Table 4:** Procedures, hospital outcomes and isolated pathogens of hospitalized patients with and without diabetes who suffered from postsurgical pneumonia according to hospitalization survival in Spain, 2001–2015.

	In-hospital mortality		P value
	Type 2 diabetes	No diabetes	
Nonmechanical ventilation, n (%)^a^^,^ ^b^	2215(40.49)	377(35.37)	0.002
Mechanical ventilation, n (%)^a^^,^ ^b^	15827(43.86)	2740(47.54)	<0.01
Thoracocentesis, n (%)	845(31.67)	136(33.66)	0.424
Pleural drainage tube, n (%)^a^^,^ ^b^	2224(36.83)	288(39.67)	0.135
Bronchoscopy, n (%)^a^^,^ ^b^	2914(35.56)	374(35.32)	0.877
Transfusion, n (%)^a^^,^ ^b^	8641(37.24)	1545(36.74)	0.539
Dialysis, n (%)^a^^,^ ^b^	3223(59.21)	533(52.56)	<0.01
Tracheostomy, n (%)^a^^,^ ^b^	8598(39.67)	1554(47.11)	<0.01
Pressure ulcers, n (%)^b^	1815(31.78)	642(34.52)	0.029
*Pseudomonas*, n (%)^a^^,^ ^b^	2189(41.66)	271(40.51)	0.571
*Staphylococcus aureus*, n (%)^a^^,^ ^b^	1532(36.71)	259(39.36)	0.191
*Streptococcus pneumoniae*, n (%)^a^^,^ ^b^	1518(26.34)	292(24.91)	0.311
*Haemophilus influenzae*, n (%)^a^	355(23.95)	65(29.68)	0.067
Aspergillosis, n (%)^a^^,^ ^b^	454(47.1)	48(41.38)	0.244
Miscellaneous, n (%)^a^^,^ ^b^	838(41.22)	128(43.54)	0.451
Respiratory culture, n (%)	1225(32.60)	205(32.54)	0.977
Emergency room admission, n (%)	23799(33.22)	5073(33.05)	0.681

p value for the difference when comparing patients with and without diabetes.

^a^ Significant association of the study variable with IHM among nondiabetic patients.

^b^ Significant association of the study variable with IHM among type 2 diabetes patients.

In both groups, IHM was higher in the older age groups, those with more coexisting conditions, those who underwent any diagnostic or therapeutic procedure (except thoracocentesis) and those who had any pathogen isolated (except *Streptococcus pneumoniae* or *Haemophilus influenzae)*.

When we compare the IHM between T2DM patients with those without T2DM according to the type of surgical procedure, we find higher figures for diabetic patients who underwent surgical procedures involving the respiratory system, nervous system and musculoskeletal/integumentary system. IHM was also higher among those who were treated with noninvasive mechanical ventilation and dialysis. However, IHM was higher among non-T2DM patients than T2DM patients in older patients (>75 years), those with higher CCI, those who had surgeries involving the cardiovascular system and those who were treated with mechanical ventilation.

Table 5 presents the results of the multivariable analysis to identify predictors of IHM after postsurgical pneumonia for patients with and without T2DM.

**Table 5:** Factors independently associated with in-hospital mortality among hospitalized diabetic and nondiabetic patients who suffered from postsurgical pneumonia according to hospitalization survival in Spain, 2001–2015.

		Type 2 diabetes OR, 95% CI	No diabetes OR, 95% CI	Total OR, 95% CI
Female sex		1.00(0.97–1.03)	1.04(0.97–1.11)	1.01(0.98–1.04)
Age groups (years)	40–54	1	1	1
	55–64	1.38(1.31–1.46)	1.41(1.19–1.66)	1,39(1,32–1,47)
	65–74	2.00(1.91–2.1)	1.85(1.59–2.16)	1,99(1,9–2,08)
	75–84	2.92(2.78–3.07)	2.83(2.42–3.3)	2,92(2,79–3,06)
	≥ 85	4.43(4.17–4.71)	3.98(3.35–4.74)	4,36(4,12–4,62)
CCI	0	1	1	1
	1	1.44(1.39–1.49)	1.41(1.29–1.54)	1.44(1.39–1.49)
	≥ 2	1.68(1.62–1.75)	1.65(1.51–1.81)	1.68(1.62–1.74)
Nonmechanical ventilation		1.27(1.19–1.35)	1.06(0.92–1.22)	1.23(1.16–1.3)
Mechanical ventilation		2.9(2.8–3.01)	3.03(2.78–3.29)	2.93(2.83–3.03)
Thoracentesis		0.90(0.82–0.98)	0.97(0.77–1.23)	0.91(0.83–0.99)
Pleural drainage tube		1.12(1.05–1.19)	1.28(1.08–1.53)	1.13(1.07–1.2)
Transfusion		1.23(1.19–1.27)	1.24(1.15–1.34)	1.23(1.19–1.27)
Dialysis		3.05(2.87–3.24)	2.49(2.16–2.87)	2.95(2.79–3.12)
*Pseudomonas*		1.15(1.08–1.22)	0.99(0.83–1.18)	1.13(1.06–1.2)
*Streptococcus pneumoniae*		0.73(0.68–0.78)	0.67(0.58–0.77)	0.72(0.68–0.76)
*Haemophilus influenzae*		0.56(0.49–0.63)	0.72(0.52–0.98)	0.58(0.51–0.65)
Aspergillosis		2.01(1.75–2.32)	1.72(1.14–2.59)	1.99(1.74–2.27)
Miscellaneous		1.21(1.1–1.34)	1.24(0.96–1.6)	1.22(1.11–1.33)
Respiratory culture		0.93(0.86–1)	0.9(0.75–1.09)	0.92(0.86–0.99)
Emergency room admission		1.35(1.3–1.4)	1.32(1.21–1.43)	1.34(1.3–1.38)
Diabetes		NA	NA	0.99(0.96–1.03)
Year		0.79(0.77–0.80)	0.76(0.73–0.81)	0.78(0.77–0.81)

CCI: Charlson comorbidity index.

NA. Not applicable

Being older, suffering more comorbid conditions according to the CCI, emergency room admission and undergoing therapeutic procedures such as dialysis, pleural drainage tube usage, any type of mechanical ventilation and blood transfusion were predictors of higher IHM in both populations. However, T2DM patients who had thoracentesis had a lower risk of IHM (OR 0.90, 95% CI 0.82–0.98).

Isolation of *Haemophilus influenza* or *Streptococcus pneumoniae* predicted a lower IHM in patients with T2DM. On the other hand, isolation of *Aspergillosis* (for both groups) or *Pseudomonas* (for those with T2DM) increased the risk of dying.

Temporal trend analysis, using multivariable adjustment, showed a significant reduction in IHM from 2001 to 2015 in T2DM (OR 0.79 95% CI 0.77–0.80) and nondiabetic patients (OR 0.76 95% CI 0.73–0.81).

Finally, we found that in our studied population, T2DM was not associated with IHM after postsurgical pneumonia (OR 0.99, 95% CI 0.96–1.03).

## Discussion

In Spain using data from the national hospital discharge database, we found higher incidence rates of hospitalization with postoperative pneumonia in patients suffering T2DM than in patients without this condition. Therefore, T2DM is a risk factor for developing postoperative pneumonia. Similar to other authors, we found that a higher prevalence of postoperative pneumonia in patients with T2DM occurred in the setting of orthopedic and spine surgery, cardiothoracic surgery and general surgery [9]. In a retrospective cohort study including patients undergoing total joint arthroplasty, the presence of diabetes was found to be a predictor for postoperative pneumonia (RR 1.2, 95% CI 0.9–1.5 for noninsulin-dependent diabetes vs no diabetes) [19]. A previous study using the American College of Surgery National Surgical Quality Improvement Program database, which controlled for demographics and other comorbidities, demonstrated that patients with diabetes undergoing lumbar fusion surgeries were at greater risk for postoperative complications, longer hospitalization times, and more readmissions [13]. This increased risk might be associated with immunosuppression caused by diabetes [20]. In the US, an observational study of 16,084 patients who underwent coronary artery bypass grafting identified seventeen preoperative risk factors associated with postoperative pneumonia, including diabetes as a comorbid disease (OR 1.26; p = 0.02) [21]. After the hepatectomy procedure, diabetes was associated with an approximately 2-fold increased risk of postoperative pneumonia. The authors concluded that diabetic patients had several mechanisms that could increase their risk of infection, including increased altered immune cell function, bacterial proliferation and changes in vascular permeability and endothelial cells [10,11]. In 2016, Miki et al. reported that after gastrectomy for gastric cancer treatment, diabetes increased the risk of postoperative pneumonia by 2.46-fold [22]. A suggested explanation is that poor glycemic control or a longer duration of diabetes could increase susceptibility to postoperative pneumonia [23].

In our investigation, we detected a significant increase in the incidence of postsurgical pneumonia in T2DM patients. We think that three possible reasons could explain this increase. First, according to several studies, Spanish T2DM patients are undergoing more complex surgeries, all of which have a high risk of postsurgical complications (coronary artery bypass graft, surgical aortic valve replacement, solid organ transplants, bariatric surgery, revision of total hip and knee arthroplasty) more frequently over time. Furthermore, the rates of these surgeries are increasing over time among T2DM patients and are higher than those among nondiabetic patients [24–28]. Second, as described in this and previous investigations, the mean age and the prevalence of concomitant conditions among T2DM patients have increased in recent decades [24–29]. Third, as suggested by Trinh et al., pneumonia is becoming better recognized in postoperative patients, resulting in an increase in the incidence [30].

T2DM was not a predictor of IHM after postoperative pneumonia. Umpierrez et al. found that incident hyperglycemia was a better predictor of IHM than a prior history of diabetes, concluding that hyperglycemia and not diabetes itself increased the risk of death [31]. Serio et al. (2013) investigated the postoperative risk of morbidity and mortality in diabetic patients versus nondiabetic patients who underwent general or vascular surgery and found that the presence of diabetes was not predictive of mortality; however, vascular surgery itself was predictive of mortality [32]. Perhaps the fact that subjects suffering diabetes were more likely to be admitted to the hospital for less severe illnesses might explain this association. Unfortunately, the CMBD does not include information on the severity of T2DM, and this variable could affect IHM after postoperative pneumonia. Furthermore, the presence of other T2DM complications, such as kidney disease, heart attack, stroke or peripheral vascular disease, has been demonstrated to increase the risk of mortality after postoperative pneumonia [9,19,21,22]. In our investigation, among T2DM patients, IHM was associated with a higher number of conditions included in the CCI (kidney disease, heart attack, stroke and peripheral vascular disease) and with undergoing dialysis during hospitalization, confirming the negative effect of T2DM chronic complications in the IHM after postoperative pneumonia.The IHM was higher in T2DM patients than non-T2DM patients who had undergone surgical procedures involving the respiratory system, nervous system, and musculoskeletal/integumentary system and those who required noninvasive mechanical ventilation or dialysis. The site or type of surgery could have more importance in the probability of developing pulmonary complications or adverse outcomes than the pulmonary condition of the patients prior to the intervention [33].

The results of Studer et al. [34] align with our findings, as they identified higher age and intraoperative red cell transfusion as predictors of IHM after postoperative pneumonia.

The negative effect of blood transfusion causing transient immunosuppression has been found in other studies. [35] Other predictors of higher postoperative mortality described by other authors include poor underlying medical conditions, prolonged mechanical ventilation, pleural effusion and emergency surgery compared with elective surgery [36–40].

We described a positive decline in IHM in addition to diabetes status. In a recent study, Chughtai et al. analyzed trends in the incidence of postoperative pneumonia from 2009 to 2013 in different surgical services and found reductions in all of them. Appropriate patient selection and optimization of preoperative management might have contributed to improved outcomes. [9]

The most relevant findings from our investigation for clinicians and researchers in the management of patients with pneumonia and diabetes is that the risk of suffering from postoperative pneumonia increases if patients also have T2DM; therefore, preventive measures must be strictly performed in these patients. Furthermore, among T2DM patients, those who were older, had more comorbid conditions or were admitted through the ER had a higher IHM; thus, rapid diagnosis and aggressive therapeutic procedures and treatments must be considered for those with these risk factors.

Further investigations should include mortality rates after hospital discharge and an analysis of the effect of diabetes treatments and controls (glycosylated hemoglobin) on the incidence and outcomes of postoperative pneumonia among T2DM patients.

There are several limitations of our investigation that must be considered.

We selected patients aged 40 years or older because, according to Spanish studies, the prevalence of T2DM is very low under this age (<1%) [41, 42]. Furthermore, even if the validity of diabetes as a discharge diagnosis had been demonstrated in previous investigations, the possibility of miscoding T1DM as T2DM is higher in younger groups [43, 44]

As occurs with any administrative database, changes in coding practices over time and coding errors may affect the validity of our results.

Previous investigations have found that the decision to admit a patient and the indication for surgery is affected by factors such as the age, dependency, comorbid conditions, mental health and severity of acute illness [45,46]. Spanish T2DM patients are older and have more comorbidities and disabilities than nondiabetic subjects and therefore may have fewer indications for surgery, so T2DM patients could be underrepresented in the study sample [47].

Another limitation is the lack of data regarding glycosylated hemoglobin measurements or blood glucose levels prior to or during hospitalization. Furthermore, other known risk factors for postoperative complications, such as antimicrobial treatments, number of days on ventilator support, and illness or disease severity are not collected in the CMBD[48].

In conclusion, T2DM patients have a higher incidence of postoperative pneumonia than those without this disease. The IHM dropped from 2001 to 2015, regardless of T2DM status, and diabetes did not predict a higher IHM after suffering postoperative pneumonia.

## Abbreviations

CCI: Charlson comorbidity index
CMBD: Spanish National Hospital Discharge Database, *Conjunto Mínimo Básico Datos*
ER: Emergency room
HAP: Hospital-acquired pneumonia
ICD-9-MC: International Classification of Diseases—Ninth Revision, Clinical Modification
IHM: In-hospital mortality
T2DM: Type 2 diabetes mellitus
VAP: Ventilator-associated pneumonia
